# Pomegranate Peel Phytochemistry, Pharmacological Properties, Methods of Extraction, and Its Application: A Comprehensive Review

**DOI:** 10.1021/acsomega.3c02586

**Published:** 2023-09-19

**Authors:** Jyoti Singh, Hamita Preet Kaur, Anjali Verma, Arshminder Singh Chahal, Kaushal Jajoria, Prasad Rasane, Sawinder Kaur, Jaspreet Kaur, Mahendra Gunjal, Sezai Ercisli, Ravish Choudhary, Mehmet Ramazan Bozhuyuk, Ebru Sakar, Neva Karatas, Melekber Sulusoglu Durul

**Affiliations:** † Department of Food Technology and Nutrition, School of Agriculture, Lovely Professional University, Phagwara, Punjab 144411, India; ‡ Department of Horticulture, Faculty of Agriculture, Ataturk University, 25240 Erzurum, Türkiye; § HGF Agro, ATA Teknokent, 25240 Erzurum, Türkiye; ∥ Division of Seed Science and Technology, ICAR-Indian Agricultural Research Institute, New Delhi 110012, India; ⊥ Department of Horticulture, Faculty of Agriculture, Igdir University, 76100 Igdir, Türkiye; # Department of Horticulture, Faculty of Agriculture, Harran University, 63290 Sanliurfa, Türkiye; 7 Department of Nutrition and Dietetics, Faculty of Health Sciences, Ataturk University, 25240 Erzurum, Türkiye; 8 Department of Horticulture, Faculty of Agriculture, Kocaeli University, 41380 Kocaeli, Türkiye

## Abstract

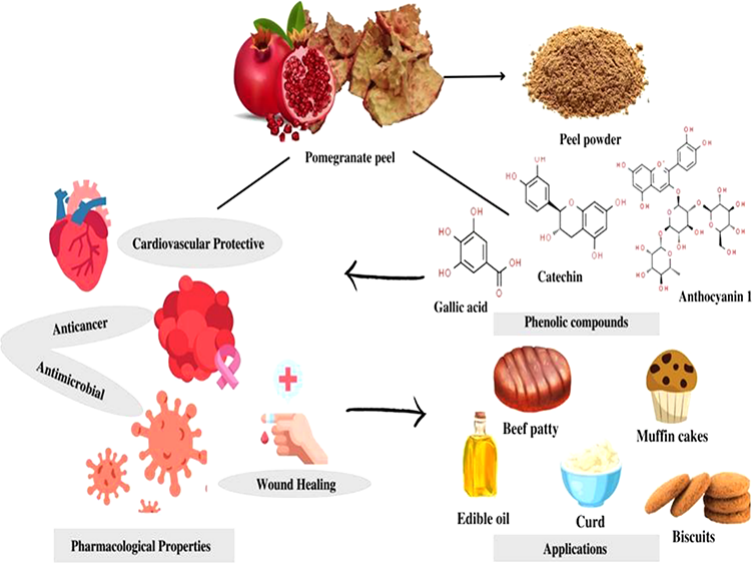

Pomegranate peel, derived from the processing of *Punica granatum L*. (pomegranate), has traditionally been considered agricultural waste. However, recent studies have revealed its potential as a rich source of bioactive compounds with diverse pharmacological effects. Pomegranate peel is a rich reservoir of antioxidants, polyphenols, dietary fiber, and vitamins, which contribute to its remarkable bioactivity. Studies have demonstrated the anti-inflammatory, cardioprotective, wound healing, anticancer, and antimicrobial properties of pomegranate peel owing to the presence of phytochemicals, such as gallic acid, ellagic acid, and punicalagin. The extraction of bioactive compounds from pomegranate peel requires a careful selection of techniques to maximize the yield and quality. Green extraction methods, including pressurized liquid extraction (PLE), ultrasound-assisted extraction (UAE), microwave-assisted extraction (MAE), and enzyme-assisted extraction (EAE), offer efficient and sustainable alternatives to traditional methods. Furthermore, pomegranate peel has been utilized in the food industry, where it can significantly enhance the nutritional value, organoleptic characteristics, and shelf life of food products. Pomegranate peel has the potential to be used to develop innovative functional foods, nutraceuticals, and other value-added products, providing new opportunities for the pharmaceutical, cosmetic, and food industries.

## Introduction

Every year, about one-third of the food produced worldwide is lost or wasted, which rises to 40% in affluent nations.^1^ The FAO Food Loss Index tracks food losses at the national level along the whole supply chain, from agricultural production to retail. According to FAO estimates, this process results in a global loss of 13.8%. Just pomegranate peel is thought to account for 1.6 billion tons of the world’s annual food waste.^1^ A member of the Punicaceae family, the pomegranate (*Punica granatum*) gets its name from the Latin word for the fruit, “Malum granatum”, which translates to “granular apple”. The pomegranate “peel” is the tough, inedible portion. The peel’s two distinct sections are the pericarp and mesocarp.^2^

India was the world’s largest pomegranate grower, with a surface area of 234,000 ha and 2.84 million metric tons of production in 2018.^3^ In 2017, the world generated an estimated 1.9 million metric tons of peel, which makes up 50% of the pomegranate fruit.^4^ Pomegranate peel is a great source of polyphenols, dietary fiber, and vitamins (Table 1), as well as other bioactive compounds. Numerous *in-vitro* and *in-vivo* studies have demonstrated that these compounds have a wide range of biological activities and health advantages, including antioxidant, anticancer, and anti-inflammatory properties.^5^ The utilization of byproducts from pomegranate processing, which are abundant in beneficial bioactive compounds, has the potential to contribute to the development of a wide range of products. These may include industrial enzymes, functional ingredients that improve the quality of food, and additives employed in the food industry to extend the shelf life of products.^6^ The current review focuses mainly on the pharmacological effects of bioactive substances found in pomegranate peel, methods for extraction of bioactive compounds, and the utilization and application of pomegranate peel in the development of value-added products.

**Table 1:** Proximate Composition and Vitamin and Mineral Content of Pomegranate Peel Powder^7−13^

**Parameter**	**Value**
**Proximate Composition** (g/100 g)	
Moisture	8.43–13.80
Protein	3.24–3.46
Fat	0.55–3.36
Crude fiber	17.43–35.19
Ash	3.35–6.07
Carbohydrates	59.52–61.34
**Vitamins** (mg/100 g)	
Vitamin A	0.16–0.18
Vitamin E	3.99–4.13
Vitamin C	12.90–13.26
Vitamin B1	0.12–0.14
Vitamin B2	0.07–0.09
**Minerals** (mg/100 g)	
Calcium	338.50–342.00
Potassium	146.40–164.30
Sodium	64.63–68.00
Phosphorus	117.90–120.00
Iron	5.93–10.25

## Pomegranate Peel Phytochemistry

**Figure 1:**
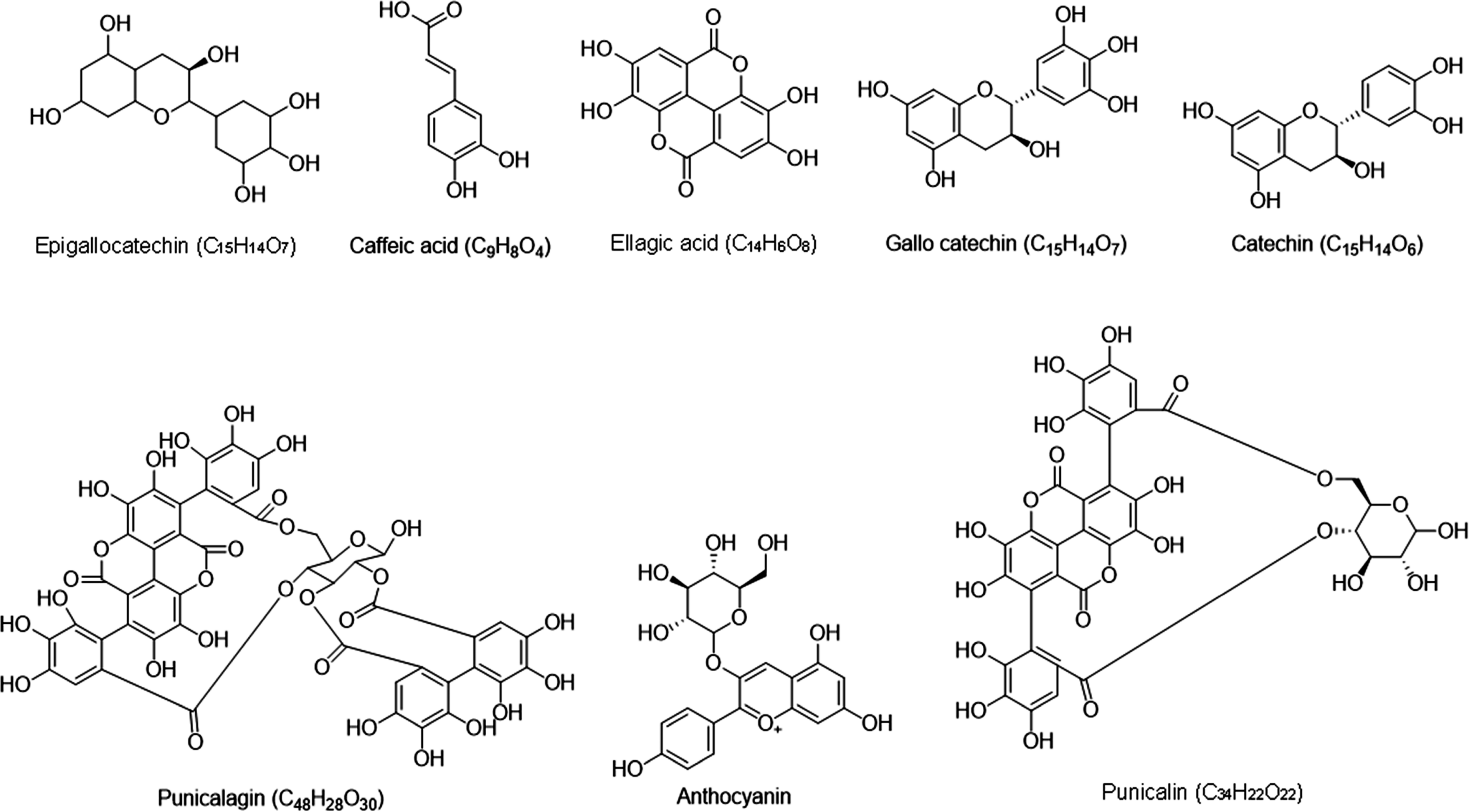
Chemical structures of the bioactive compounds present in pomegranate peel.

The term “phytochemicals” refers to substances derived from plants that are not necessary for human sustenance but have a number of bioactive attributes that enhance human health.^14^ Pomegranate peel, which is frequently seen as agricultural waste, serves as a great source of antioxidants and a variety of phytochemicals. Gallo tannins, ellagic acid, punicalins, punicalagins, and gallic acid are some of the major phytochemicals present in pomegranate peel, as shown in Figure 1.^15^ El-Hamamsy and El-khamissi^16^ revealed that pomegranate peel extract (PPE) is rich in several phytochemicals, including tannins, steroids, phenolics, alkaloids, flavonoids, terpenoids, and saponins. Nine polyphenolic constituents, including *p*-coumaric acid, syringic acid, benzoic acid, ellagic acid, caffeic acid, cinnamic acid, protocatechuic acid, isoferulic acid, and quinic acid, were identified and quantified in the extracts using HPLC.

The antimicrobial properties of PPE are attributed to its phytochemical composition, which includes gallic acid, punicalagin, rutin, resorcinol, quercetin, and syringic acid.^17^ The type of solvents used and the extraction processes determine the quantity of phenolic compounds, their capacity for scavenging free radicals, the antibacterial activity, and other biological functions of the pomegranate peels.^18^Table 2 represents quantitative analysis of some phytochemical compounds found in pomegranate peel.

**Table 2:** Quantitative Analysis of Phytochemicals Present in Pomegranate Peel^8,12,19−21^

**Compound^a^**	**Conc(mg/100 g)**
Total phenolic content (GAE)	4892.00–6138.20
Total flavonoid content (QE)	529.50–862.50
Ellagic acid	44.19–52.03
Catechin	850.00–892.00
Gallic acid	125.80–128.10
*p*-Coumaric acid	14.00–17.64
Quercetin	5
Ferulic	5.00–6.11

a GAE, gallic acid equivalent; QE, quercetin equivalent.

## Pharmacological Properties of Pomegranate Peel

**Figure 2:**
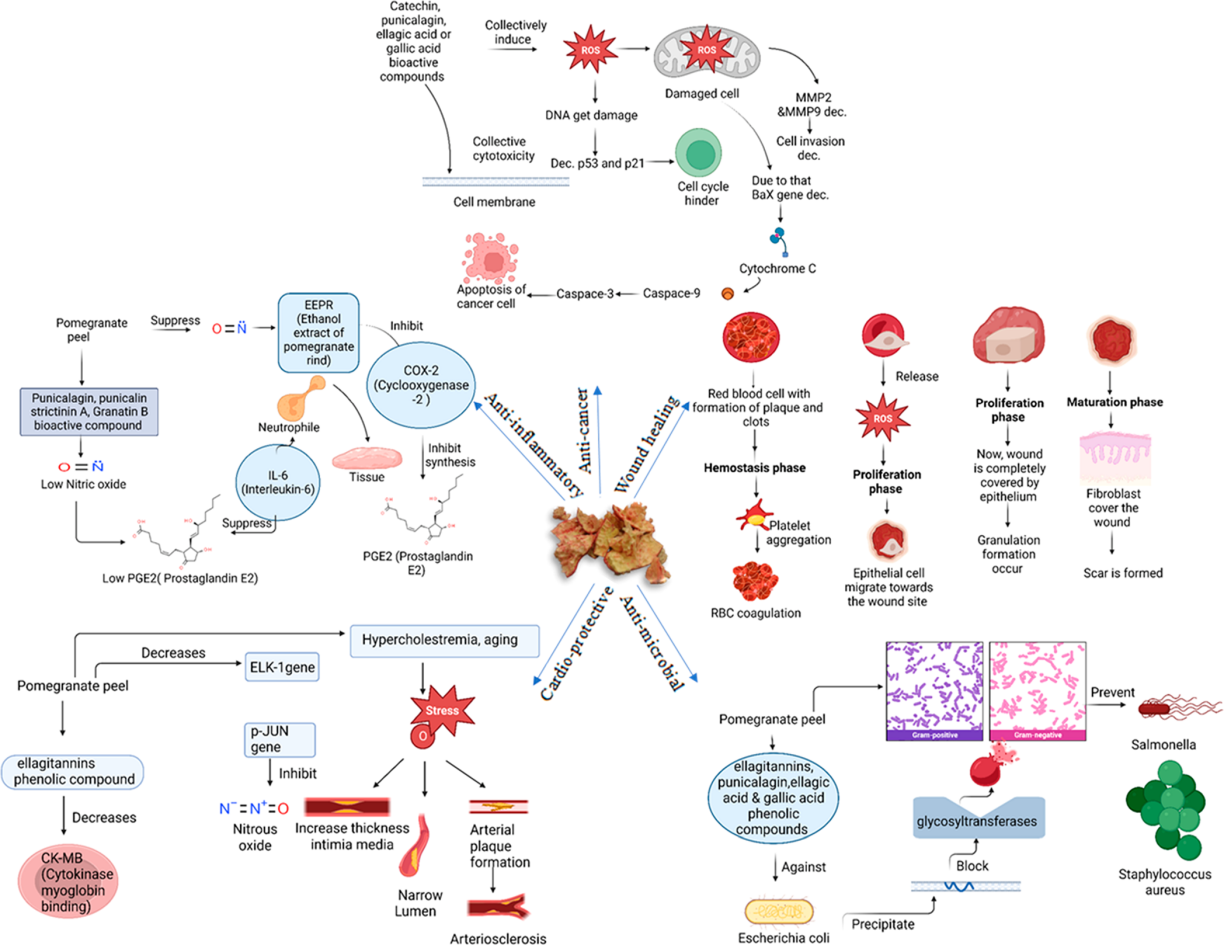
Pharmacological properties of the pomegranate peel.

Numerous phytochemicals, including catechins, flavonoids, tannins, gallic acids, ellagic acid, and anthocyanins, have been associated with the medicinal potential of pomegranate peel.^22^ According to various studies, pomegranate peel has a higher concentration of biologically active components than the fruit’s other edible portions.^23−26^ The punicalagin (α and β), ellagic acid, and ellagic acid-hex of pomegranate peel are all capable of causing cell cycle arrest in the S-phase and increase cells with fragmented DNA, which indicates induction of apoptosis.^27^ A notable increase in the levels of superoxide dismutase (SOD), glutathione (GSH), and catalase (CAT) and a simultaneous decrease in malondialdehyde (MDA) levels in the bloodstream have been observed upon administration of PPE.^28^Table 3 covers the cardio-protective, anticancer, antimicrobial, wound healing, and anti-inflammatory properties of pomegranate peel. Figure 2 illustrates the pharmacological properties of pomegranate peel.

**Table 3:** Pharmacological Activities of Pomegranate Peel

**Pharmacological activity**	**Plant part**	**Model (cell/animal/humans)**	**Dosage/conc**	**Effects**	**Ref**
Cardio-protective	Pomegranate extract capsule	Human intervention study (*in vivo*)	Walnuts (30 g/day)	Urolithin metabotype A showed positive correlation with ApoA-I. Urolithin metabotype A phenotype protects against CVDs compared to urolithin metabotype phenotype B.	(32)
			Pomegranate extract capsule (450 mg/day)		
			Mixed nuts (30 g/day)		
	Hydroethanolic PPE extract	Apoe^–/–^ mice (*in vivo*)	200 mg/kg	Improved metabolic profile (decreased total cholesterol, triglycerides, plasma insulin, blood glucose levels, improved glucose tolerance). Reduction of proinflammatory cytokines and plaque necrosis.	(33)
	Pomegranate peel polyphenols	Human hepatic L-02 cells (*in vitro*)	10, 20, and 40 μg/mL (pomegranate peel polyphenols, punicalagin, pomegranate ellagic acid)	Decreased total cholesterol and increased total bile acid content. Up-regulation of PPARγ, ABCA1, and CYP7A1 mRNA expression.	(34)
	PPE	Wistar albino rats (*in vivo*)	50 or 100 mg/kg body weight (PPE), 1 mg/kg body weight (ellagic acid), and 7 mg/kg body weight (punicalagin)	Reduced TC, TAG, LDL cholesterol, VLDL cholesterol, atherogenic index of plasma, atherogenic coefficient. Improved activity of GR, SOD, CAT, and GSH. Elevated serum PON1 activity.	(35)
Anticancer	Pomegranate peel polyphenols	Human hepatoma cells HepG2 (*in vitro*)	50 and 100 μM (punicalagin and ellagic acid)	Reduced the HepG2 cells survival rate. Punicalagin and ellagic acid arrested the cells in the S phase and G0/G1 phase of the cell cycle resulting in apoptosis. Increased caspase 3/9 activity and apoptosis related genes.	(37)
	PPE	MCF-7 and MDA-MB-231 cells (*in vitro*)	Punicalagin concentration (0, 12.5, 25, 50, 100 μM)	Punicalagin (>50 μM) inhibited viability, migration, and invasion of MDA-MB-231 and MCF-7 cells. A substantial decrease in the expression of GOLPH3, MMP-9, MMP2, and N-cadherin and an increase in the expression of E-cadherin were observed.	(38)
	PPE	DU145, PC3, TRAMP-C1 cell lines (*in vitro*)	0, 12.5, 25, 50, 100, and 200 μg/mL	PPE suppressed growth on prostate cancer cells, increased expression of pro-apoptotic Bax, and decreased expression of antiapoptotic Bcl2. Up-regulation of MMP2/9 expression and mitochondrial mediated apoptosis in TRAMP-C1.	(39)
	PPE	BCPAP and TPC-1 cell lines (*in vitro*)	0, 12.5, 25, 50, 100, 200 μg/mL	PPE considerably reduced proliferation in cell lines, thus exhibiting cytotoxic and cytostatic activity. Concentration-dependent apoptosis was induced in cancer cell lines. PPE reduced the mitochondrial membrane potential, thereby inducing apoptosis.	(40)
	PPE	Female BALB/c nude mice (*in vivo*)	125 mg/kg and 62.5 mg/kg body weight	Tumor growth was inhibited by preventing metastasis, promoting apoptosis, and reducing the proliferation of cells.	
Antimicrobial	Pomegranate peel polyphenols	*Ralstonia solanacearum* model strain GMI1000 (*in vitro*)	0, 5, 10, and 15 μg/mL	Growth curve was steady in treated cultures. Cell wall and cell membrane were detached. Bacterial motility was reduced. Attachment of punicalagin with functional domains of PhcA resulted in disarranged network eventually leading to bacterial damage.	(48)
	PPE	Wistar rats (*in vivo*)	125, 250, and 500 mg/kg/d BW	Decreased the growth of *Candida albicans.* Compared to nystatin, pomegranate peel exhibited 100% efficacy at all doses. Preserved the natural structure of epithelium, muscular core, and lamina propria in tongue.	(49)
	PPE	Swiss albino mice (*in vivo*)	100 μL (300 mg/kg)	ELISA revealed a gradual reduction in *Giardia* antigen in the feces of mice treated with PPE. A decrease in the cyst formation with a simultaneous increase in cure rate was observed in the experimental group.	(50)
Wound healing	Pomegranate pele extract	Rats (*in vivo*)	5 g PPE/100 g gel	Increase in collagen content, hydroxyproline levels, wound contraction, expression of EGF, VEGF, and TGF- β1, epithelialization, and granulation was observed in the treatment group.	(53)
Anti-inflammatory	Ethanolic PPE	Swiss Webster mice (*in vivo*)	240 and 480 mg/kg/d (Doses-1 and Doses-2, respectively)	Reduction in COX-2 and iNOS expression via inhibition of the NF-κB pathway was reported.	(58)
	Pomegranate peel polyphenols	RAW264.7 macrophage (*in vitro*)	1, 10, 100 μg/mL (pomegranate peel polyphenols)	Test polyphenols down-regulated LPS induced NO and PGE2 generation. Decreased pro-inflammatory cytokines and inhibited MAPKs pathway.	(59)
			1, 10, 50 μM (punicalagin and ellagic acid)		

### Cardio-protective Properties

Worldwide, cardiovascular diseases continue to be the main cause of mortality.^29^ Numerous disorders, including obesity, nonalcoholic fatty liver disease (NAFLD), cardiovascular diseases, insulin resistance, atherosclerosis, and hypertension, have been shown to be primarily caused by the dysregulation of lipid metabolism.^30^ With diverse chemical configurations, plant-based bioactive compounds are known to possess potent cardioprotective properties.^31^

The utilization of ellagitannin-metabolizing phenotypes as prospective biomarkers for cardiovascular risk was examined by Selma et al.^32^ Three trials were carried out, which included consumption of walnuts (30 g/day), pomegranate extract (450 mg/day), and mixed nuts (30 g/day). The findings revealed that those with the urolithin metabotype B phenotype were more likely to develop cardiovascular diseases (CVDs), especially those who were overweight or obese and had metabolic syndrome. The urolithin metabotype A phenotype, on the other hand, appeared to provide defense against CVD risk factors. This study also explored the association between specific biomarkers for CVD risk and isourolithin-A and urolithin-B excretion. Urolithin-A exhibited a favorable correlation with the antioxidant and anti-inflammatory compound ApoA-I. This study implies that urolithin metabotypes may be effective tools for determining CVD risk. However, further studies with larger cohorts are required to confirm these results.

Manickam et al.^33^ demonstrated that Apoe^–/–^ mice (*in vivo*) fed a Western diet did not develop advanced atherosclerosis. This was accomplished by administering a standardized hydroethanolic extract of pomegranate peel with a high polyphenol content. An *ad libitum* Western-style diet rich in fat and cholesterol was provided to the animals for 12 weeks, along with daily oral gavage administration of either 200 mg/kg PPE or vehicle (water). PPE-treated mice showed considerably reduced blood glucose levels, improved glucose tolerance, decreased plasma insulin levels, and significantly lower plasma total cholesterol and triglyceride levels. Furthermore, reduced plasma levels of the proinflammatory cytokine TNF and higher production of the anti-inflammatory cytokine IL-10 showed that systemic inflammation was reduced in PPE-fed mice. PPE supplementation resulted in a significant reduction in plaque necrosis, a crucial factor linked to plaque rupture, and clinical signs of myocardial infarction and stroke in humans. Lower levels of F4/80 + macrophages and CD3+ T cells were observed within the atherosclerotic lesions, whereas higher levels of smooth muscle cells were observed. Higher lesional macrophage efferocytosis efficiency and higher expression of the Mertk efferocytosis receptor were both related to a reduction in plaque necrosis. Hence, the bioactive compounds in PPE, such as gallic acid, chlorogenic acid, *p*-coumaric acid, caffeic acid, punicalagin A, and punicalagin B (UPLC analysis), act as potent antioxidant and cardioprotective components.

Lv et al.^34^ investigated the effects of pomegranate peel polyphenols, its primary component punicalagin, and pomegranate ellagic acid (a punicalagin metabolite) on lipid accumulation and cholesterol metabolism mediated by the PPARγ-ABCA1/CYP7A1 cell signaling system in hepatic L-02 cells (*in vitro*). These findings demonstrated that pomegranate peel polyphenols, punicalagin, and pomegranate ellagic acid at various concentrations (10, 20, and 40 μg/mL) increased the total bile acid content and reduced the total cholesterol content, resulting in lipid-lowering effects. The assessed polyphenols increased the relative mRNA expression of ATP-binding cassette transported A1 (ABCA1), cholesterol 7-hydroxylase (CYP7A1), and peroxisome proliferator-activated receptor γ (PPARγ). Cells treated with each of the compounds examined at various concentrations exhibited a dose-dependent behavior. A notable negative correlation between total cholesterol and the mRNA levels of PPARγ, ABCA1, and CYP7A1 was found in L-02 cells after exposure to test compounds, while a considerable positive correlation was found between total bile acid levels and hepatic expression of PPARγ, ABCA1, and CYP7A1. These findings suggest that pomegranate peel polyphenols, punicalagin, and pomegranate ellagic acid might control PPARγ, ABCA1, and CYP7A1 expression upstream at the transcript and protein levels, thereby activating PPARγ, ABCA1, and CYP7A1 cell signaling systems and improving cholesterol metabolism in L-02 cells.

Soliman et al.^35^ examined the effects of pomegranate peel, in contrast to the pure form of its polyphenols (ellagic acid and punicalagin), on high-fat diet (HFD)-induced dyslipidemia in male albino rats (*in vivo*). Oral administration of PPE (50 or 100 mg/kg body weight), ellagic acid (1 mg/kg body weight), or punicalagin (7 mg/kg body weight) for 6 weeks was performed in conjunction with a regular diet or after inducing hyperlipidemia. Compared to the HFD group, oral administration of PPE, ellagic acid, and punicalagin significantly reduced the concentrations of total cholesterol (TC), triacylglycerol (TAG), low-density lipoprotein cholesterol, and very low-density lipoprotein cholesterol and decreased the atherogenic index of plasma, the atherogenic coefficient, Castelli’s risk index I and II, and the malondialdehyde levels. The activities of glutathione reductase (GR), superoxide dismutase (SOD), and catalase (CAT) were considerably enhanced by ellagic acid, whereas the levels of glutathione (GSH), GR, and SOD were significantly elevated upon punicalagin administration. Following PPE treatment (100 mg/kg of body weight), a substantial increase in serum paraoxonase 1 (PON1) activity was observed. TNF-α levels increased considerably after PPE treatment in the hyperlipidemic groups at both dosages.

These studies highlighted how ellagic acid, punicalagin, and other bioactive components in pomegranate peel could have positive impacts on cardiovascular health. Antioxidant, anti-inflammatory, lipid-lowering, and cardioprotective actions are among the pharmacological traits of these substances. Both *in-vivo* and *in-vitro* studies have shown that they enhance antioxidant capacity, decrease atherosclerotic plaque formation, and improve lipid metabolism in addition to reducing systemic inflammation. Therefore, it can be implied that pomegranate peel and its bioactive compounds have potential as natural alternatives for reducing cardiovascular risk factors.

### Anticancer Properties

Uncontrolled cell division characterizes cancer, a genetic ailment. Phytochemicals are ingestible components of plants that have cancer-fighting and cancer-preventive properties.^36^

Li et al.^37^ examined the effects of punicalagin and ellagic acids on the cell cycle, apoptosis, reactive oxygen species (ROS), and mitochondrial trans in HepG2 cells (*in vitro*). The experimental group received final punicalagin and ellagic acid concentrations of 50 and 100 μM. Although L-02 cells (normal liver cells) were unaffected, punicalagin and ellagic acid significantly reduced the survival rate of HepG2 cells (hepatoma cells) in a dose- and time-dependent manner. The punicalagin-induced inhibition of HepG2 cell growth was more potent than the ellagic acid-induced inhibition. As both punicalagin and ellagic acid could stop HepG2 cells in the S phase and G0/G1 phase, respectively, they could potentially restrict the proliferation of HepG2 cells. Both punicalagin and ellagic acid promoted cell death in a dose-dependent manner, but punicalagin had a larger effect on apoptosis than did ellagic acid. To coordinate apoptotic reactions, the caspase protease family is crucial. An increase in ROS levels and caspase-3 and caspase-9 activities was observed. The expression of apoptosis-related proteins such as P53, Bax, and cytochrome c was considerably higher in the treatment group than in the control group.

Pan et al.^38^ determined the effect of punicalagin on cellular processes within breast cancer cells (*in vitro*). CCK-8, wound healing, and Transwell assays were used to measure the effect of different punicalagin concentrations (0, 12.5. 25, 50, and 100 μM) on the viability, migration, and invasion of MCF-7 and MDA-MB-231 cells. Following transfection of Golgi phosphoprotein 3 (GOLPH3) into cells with or without punicalagin treatment, the expression level of GOLPH3 was assessed by qRT-PCR (quantitative real-time polymerase chain reaction) and Western blotting. Both MCF-7 and MDA-MB-231 cells had lower viability at punicalagin concentrations over 50 μM, and cell migration and invasion exhibited a similar trend. MCF-7 and MDA-MB-231 cells were increased by GOLPH3 overexpression. Punicalagin substantially decreased the expression of GOLPH3 in the MCF-7 and MDA-MB-231 cells. Additionally, the expression of EMT-related proteins was examined. The findings showed that treatment with punicalagin decreased the expression of MMP-2, MMP-9, and N-cadherin, while increasing the expression of E-cadherin in MCF-7 and MDA-MB-231 cells. As a result, a potential link between punicalagin and GOLPH3 was identified, which revealed that by controlling the expression of GOLPH3, punicalagin influences the EMT process of breast cancer as well as changes in cell phenotype.

Deng et al.^39^ examined the impact of PPE on prostate cancer cells’ (DU145, PC3, and TRAMP-C1) apoptosis and metastasis, as well as the underlying mechanism. PPE was administered at various concentrations (0, 12.5, 25, 50, 100, and 200 mg/mL). In a time- and concentration-dependent manner, PPE effectively suppressed TRAMP-C1 cell proliferation. DU145 and PC3 cell growth did not decrease when exposed to low doses for 24 h, but higher concentrations and longer treatment times effectively suppressed the cell growth. The findings revealed that treatment with PPE boosted the expression of pro-apoptotic Bax and cleaved caspase 3 while decreasing the expression of antiapoptotic Bcl2. After treatment with PPE, the ROS levels in TRAMP-C1 cells significantly increased. These findings suggest that the mitochondria-mediated apoptotic pathway may be the mechanism by which PPE induces apoptosis in TRAMP-C1 cells. Treatment with PPE considerably reduced the capacity of TRAMP-C1 cells to migrate and invade. Additionally, PPE treatment increased the level of TIMP2 expression and decreased MMP2 and MMP9 expression in TRAMP-C1 cells.

Li et al.^40^ examined the anticancer potential of PPE against thyroid carcinoma *in vitro* (0, 12.5, 25, 50, 100, and 200 μg/mL) and *in vivo* (125 and 62.5 mg/kg body weight) assays. BCPAP and TPC-1 cell lines were used to evaluate the efficacy of PPE in thyroid cancer. PPE effectively reduced the proliferation of both thyroid cancer cell lines. These findings indicate the cytostatic and cytotoxic effects of PPE on thyroid cancer cells. PPE caused concentration-dependent apoptosis of BCPAP and TPC-1 cells. Additionally, PPE may lower mitochondrial membrane potential, suggesting that it may cause apoptosis through a mitochondria-mediated apoptotic pathway. PPE significantly reduced tumor development in a BCPAP-bearing mouse model by lowering cell proliferation, inducing apoptosis, and preventing metastasis.

The presence of ellagic acid and punicalagin, which can cause cancer cells to undergo apoptosis, and other ellagitannins in pomegranate peel has been proven to have anticarcinogenic characteristics. The up- and down-regulation of Bax are among the numerous mechanisms at play. Cancer cells die due to Bax activation’s promotion of mitochondrial membrane permeabilization, which causes the release of the apoptotic component cytochrome c.^41^ Cytochrome c may affect cell apoptosis, thus influencing tumor growth.^42^ Punicalagin promotes apoptosis while suppressing cell proliferation.^43^

### Antimicrobial Properties

According to phytochemical investigations, the pomegranate peel contains potent inhibitors, such as flavonoids and phenolic compounds.^44^ It is well established that various polyphenolic compounds can work synergistically to inhibit the growth of microorganisms. These compounds function as anti-infectives by forming complexes with extracellular and soluble proteins found in microbial cell walls, which precipitate membrane proteins and inhibit enzymes such as glycosyl transferases, thereby disintegrating the microorganisms.^45,46^*Salmonella* and Gram-positive *S. aureus* are strongly inhibited by PPEs, which have a strong antimicrobial effect on all bacteria. This is based on prior studies that discovered high quantities of tannins in PPE, which are highly efficient against some strains of the bacterium *Staphylococcus aureus*.^47^

Chen et al.^48^ evaluated the antibacterial activity of pomegranate peel against the plant pathogen *Ralstonia solanacearum (in vitro)*. Bacterial cells were exposed to different dosages (0, 5, 10, and 15 mg/mL) of pomegranate peel polyphenols. HPLC analysis revealed the presence of punicalagin, ellagic acid, catechin, gallic acid, chlorogenic acid, and epicatechin in the pomegranate peel. Compared to the control, the bacterial cultures containing polyphenols added to them showed a more gradual growth curve. The cytoplasmic membrane and cell wall were detached from the bacteria that received treatment. Furthermore, they significantly reduced the bacterial motility. The smallest swimming diameter was observed in the group treated with 5 mg/mL pomegranate peel polyphenols. When the integrity of the cell membrane was compromised, punicalagin was able to bind to the functional domains of the bacterial transcriptional regulator PhcA, causing the regulatory network to become disorganized and eventually causing bacterial impairment.

Bassiri-Jahromi et al.^49^ analyzed the antifungal activity of PPE against oral candidiasis and compared it to that of nystatin using Wistar rats (*in vivo*). Different doses of PPE (125, 250, and 500 mg/kg/day BW) and nystatin (10000 U/kg/day BW) were administered as part of the treatment. Irrespective of PPE concentration, a significant decrease in the growth of *C. albicans* was observed 15 days after treatment. The PPE extract exhibited a 100% cure at all doses, whereas nystatin had 80% efficacy. After treatment, the histological and morphological features revealed the natural structure of the filiform papillae, epithelium, muscular core, and lamina propria in the tongues of rats. This indicated that PPE treatment preserved the normal structure of the tongue in rats despite their immunosuppressed stage. This implies the presence of bioactive compounds in PPE that have antifungal effects against *Candida albicans*. However, these substances require further identification and characterization to establish their mechanisms of action.

Al-Megrin^50^ evaluated the effectiveness of PPE (300 mg/kg) against giardiasis in infected Swiss albino mice (*in vivo*). One hundred microliters of the extract was administered to the experimental group. The ELISA test showed a steady decrease in the amount of *Giardia* antigen in the feces of the experimental groups treated with PPE compared with the control groups. The experimental group showed a decreased level of cyst formation. Furthermore, the cure rate in the experimental group was significant. These results indicate that PPE possesses potential antimicrobial properties. Nevertheless, further investigation is required to determine the efficacy and safety of this extract.

These studies suggest that PPE contains bioactive substances such as flavonoids and phenolic compounds that have strong antimicrobial effects against bacteria, fungi, and parasites. These substances have the potential to function as natural antibacterial agents by rupturing cell membranes and blocking vital enzymes.

### Wound Healing Potential

Restoration of diseased or damaged tissues is a complicated process that involves a series of well-structured biochemical and cellular activities. Homeostasis, inflammation, proliferation, and remodeling are the four meticulous and programmed phases.^51^ Pomegranate peel contains significant levels of polyphenols, including gallic acid, ellagic tannins, and ellagic acid. Due to the presence of polyphenols and flavonoids, pomegranate peel possesses wound healing properties.^52^

Karim et al.^53^ examined the clinical, biochemical, and histological effects of Saudi PPE applied to experimentally produced full-thickness skin wounds in diabetic rats (*in vivo*). Vehicle gels and Saudi PPE (5 g of extract per 100 g of gel) were prepared. The experiment was conducted over a 21 d period. Compared with the diabetic control group, the Saudi PPE gel-treated group showed much higher wound contraction. The collagen content in diabetic wounds increased as a consequence of the elevated levels of mean hydroxyproline in the treated group. Transforming growth factor beta 1 (TGF-β1) expression in the wound tissues of the rats in the treatment group was higher than that in the vehicle group on the 14th day. Collagen production and breakdown are mediated by TGF-β1. The vascular endothelial growth factor (VEGF) tends to affect pathological events, including tissue healing, which involves neovascularization and altered vascular permeability. The epidermal growth factor (EGF) stimulates epithelial cell mitosis and chemotaxis, thereby enhancing epithelialization. Saudi PPE-treated rats showed a noticeable increase in the levels of VEGF expression and EGF content. Histological findings showed a considerable increase in the fibroblast creation rate, epithelialization, and granulation in the treated group. Secondary metabolites found within pomegranate peel, such as flavonoids and phenolic acids, play an active role in wound healing.

Topical application of the pomegranate peel to wounds may accelerate the healing process. This is because using pomegranate peel strengthens granulation tissues, which aids in wound healing.^54^ Therefore, bioactive compounds from pomegranate peel show potential for wound healing by promoting wound contraction, collagen production, and expression of growth factors.

### Anti-inflammatory Properties

Inflammation is a localized reaction of living mammalian tissues to damage.^55^ White blood cell activation, immune system chemical release, creation of inflammatory mediators, and release of prostaglandins are all components of inflammatory processes.^56^*Punica granatum* peels have long been employed in a variety of diseases treatments such as inflammation.^57^

Kusmardi et al.^58^ examined the inflammatory effects of ethanolic PPE on the colon of mice (*in vivo*) via an inflammatory route that lowers the inflammation score in the animal models of chronic inflammation brought in by dextran sodium sulfate (DSS). The ethanolic extract was rich in ellagic acid; therefore, it was chosen for the experiment. Ethanolic PPE was administered at two concentrations {240 mg/kg/day (DOSES-1) and 480 mg/kg/day (DOSES-2)} over a period of 42 days. Ethanolic PPE could reduce the levels of COX-2 (cyclooxygenase-2) and inducible nitric oxide synthase (iNOS). The enzymes COX-2 and iNOS are crucial during the inflammatory processes. The p105 protein, which is the cytoplasmic progenitor of the p50 protein, interacts with ellagic acid present in PPE, which prevents the production of protein p50 and nuclear factor kappa B (NF-κB), which is one of the key transcription factors involved in mediating inflammatory responses, and is unable to achieve the transcription of inflammatory response genes.

Du et al.^59^ evaluated the anti-inflammatory effects of pomegranate peel polyphenols at different concentrations (1, 10, and 100 μg/mL) in RAW264.7 macrophages (*in vitro*) and investigated the connection between specific components such as punicalagin (1, 10, and 50 μM) and ellagic acid (1, 10, and 50 μM) and systemic inflammation. In a dose-dependent manner, the three test polyphenols substantially inhibited lipopolysaccharide (LPS)-induced NO and PGE2 generation. Similarly, the iNOS and COX-2 mRNA levels were inhibited. The production of proinflammatory cytokines, including tumor necrosis factor α (TNF-α), interleukin-1 beta (IL-1β), and interleukin-6 (IL-6), was significantly decreased in the ellagic acid, punicalagin, and pomegranate peel polyphenol pretreatment groups (intermediate- and high-dose groups). The mRNA expressions of TNF-α and IL-1 mRNA could both be considerably decreased by polyphenols at each concentration. However, with respect to IL-6 mRNA expression, polyphenol levels were reduced, particularly at intermediate and high concentrations. In macrophages, MAPKs, including P38, ERK, and JNK, significantly affect the generation of inflammatory mediators. The expression of p-ERK/ERK after pretreatment with the test polyphenols was considerably down-regulated, and phosphorylation of MAPKs was noticeably suppressed.

These findings provide strong evidence that PPE, which is abundant in polyphenols, including punicalagin and ellagic acid, has powerful anti-inflammatory activities. They reduce proinflammatory cytokines, modify MAPK signaling, and inhibit COX-2 and iNOS production, all of which provide evidence for their potential as potent anti-inflammatory compounds.

## Extraction Techniques

Extraction is isolating and getting the preferred constituent or group of components from a plant’s raw material by employing analytical techniques.^60^ The techniques employed to extract phenolic compounds vary from traditional to green technologies. Traditional extraction methods are characterized by the utilization of substantial volumes of extraction solvents and labor-intensive manual processes that are highly dependent on the researcher; consequently, these methods are not ideally consistent. Green extraction methods were developed to overcome the limitations posed by traditional methods.^61^ Faster extraction rates, more efficient energy use, increased mass and heat transfer, smaller equipment, and fewer processing steps are the goals of these unconventional methods.^62^

### Traditional Methods

#### Solvent Extraction

In most studies, PPEs are obtained using the traditional solvent extraction approach.^63^ The pomegranate peel is treated with solvents such as methanol, chloroform, acetone, water, ethanol, and ethyl acetate to extract antioxidants. The traditional extraction technique often uses methanol or methanol mixed with additional organic solvents.^64^

The effects of several solvents, such as water, methanol, and ethanol, employed to extract phenolic components from pomegranate peel (Helow variety) were assessed, and it was concluded that aqueous peel extract exhibited a better capacity for extraction and higher total phenolic content than methanol and ethanol PPEs.^65^ Another study assessed and identified the extraction solvents (water, 70% ethanol, and ethanol p.a.) that produce high levels of antioxidant components from PPE, including phenolics and total flavonoids. Based on the measurements made using the DPPH and CUPRAC techniques, the 70% ethanol extract of pomegranate peel has a higher antioxidant capacity than the water and ethanol p.a. extracts.^66^ Efficiency increases with temperature, as heat opens up cell walls, increases the solubility and diffusion coefficient of substances to be extracted, and decreases viscosity, which makes it easier for solvents to pass through the solid substrate mass. However, the total amount of polyphenols and flavonoids decreases at temperatures above 40 °C, perhaps due to their destruction.^67^

#### Soxhlet Extraction

This method is still extensively used to extract several natural bioactive substances, particularly phenolic compounds, from various sources. Pretreated plant material is subjected to various solvents, including water, ether, hexane, chloroform, benzene, methanol, acetonitrile, and ethanol, which capture the desired molecules: polyphenols.^68,46^

**Figure 3:**
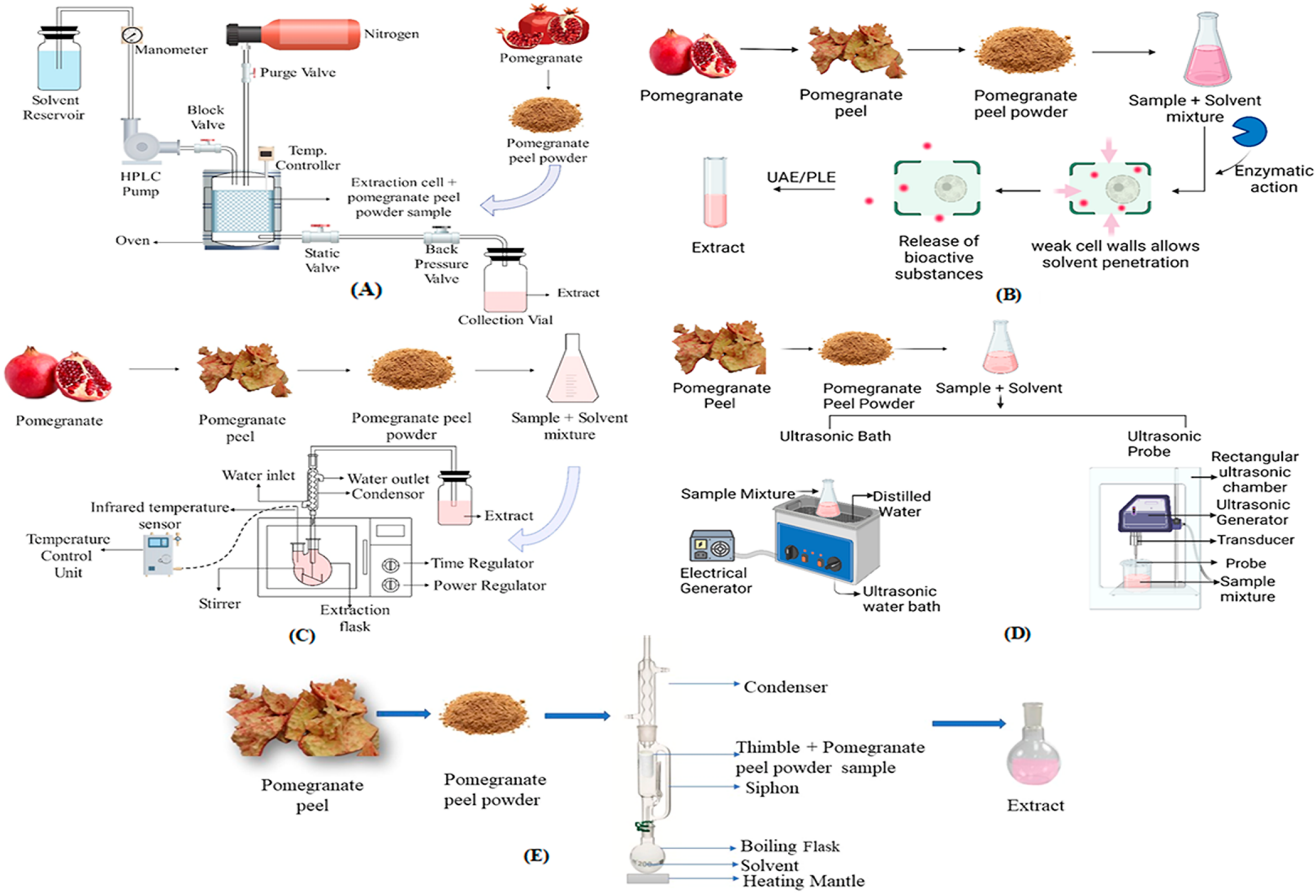
Methods of extraction representing (A) PLE, (B) EAE, (C) MAE, (D) UAE, and (E) Soxhlet extraction.

The sample was placed in a thimble attached to a solvent-filled round-bottom extraction flask and condenser of a Soxhlet apparatus, as shown in Figure 3E.^69^ The reflux operation was repeated several times before extraction was complete.^70^ The components were separated throughout the extraction process based on their polarity and solvent. The least polar compounds of the dry material were extracted using the least polar solvents, such as petroleum ether. In contrast, a steady rise in polarity up to water results in the extraction of the most polar compounds.^71^

The Soxhlet extraction process is considered because of its enhanced simplicity. However, this method has certain limitations. It utilizes more samples, takes comparatively longer duration for extractions, consumes large amounts of solvent, and loses much thermal energy in the process.^72^

### Green Technologies

#### Pressurized Liquid Extraction (PLE)

This extraction technique utilizes solvents under pressure and at elevated temperatures, increasing efficiency.^60^ Pressurized liquid solvents are used in this method, which operates at pressures of >4 MPa and temperatures ranging from mild to high. High pressure helps drive the solvent into the pores of the matrix, which improves the ability of the target compounds to dissolve.^73^ The extraction process involves placing the sample in a stainless-steel extraction cell after being combined with sea sand. To prevent suspended particles from entering the collection bottles, a cellulose filter in the shape of a circle was placed at the base of the extraction cell.^74^ After the extraction cell was heated, and an HPLC pump was used to pressurize the system. Once the desired pressure is attained by shutting off the solvent outflow with the blocking valve, the pressure is maintained for static extraction until system equilibrium is reached. The static and back-pressure valves were opened and precisely regulated to maintain the required system pressure. The solvent is then pumped, penetrating the vegetal matrix and beginning the dynamic extraction process, which extracts solvent-soluble bioactive compounds at a constant flow rate. The extract is collected in collecting vials (Figure 3A) after nitrogen gas has been purged.^75−77^ As water and/or ethanol are GRAS (generally acknowledged as safe) solvents, employing PLE with them is even more promising.^78^

In one study, the extraction of phenolic compounds from pomegranate peels using a combination of ultrasonic and pressurized liquid extraction (UAPLE) was assessed (*Punica granatum L*.). According to the results, the extraction of phenolic compounds was strongly influenced by the temperature, the solvent, and their interactions. Large-particle extraction yields were more affected by ultrasound, and an intermediate ultrasound power delivered the maximum result. Thus, this combination emerged as an immaculate, effective, and green technology for extraction.^79^

The type of solvent (centered on polarity), the state of the solvent (liquid, gas, or both, based on the pressure as well as temperature), the rate of flow of the pressurized fluid (based on mass transfer and kinetics), and the pH of the medium are the principle extraction parameters considered in pressurized fluid processing.^80^ Compared to the extracts obtained from traditional methods, the purity of the extracts is one of the positive states of pressurized liquid extraction, as per many studies.^81−84^ However, the main drawbacks of this method include limited analyte selectivity during extraction, the prevalence of interferents during extraction, an elevated level of extract dilution, particularly when employing multiple cycles, and the requirement for expensive, advanced instruments.^61^ Anthocyanin, flavonoids, and other compounds have been successfully extracted from various fruits and vegetables using this technique. Additionally, owing to its processing rate and reduced solvent usage, this method was shown to be a more desirable procedure within the food industry.^85^

#### Ultrasound-Assisted Extraction (UAE)

This method is based on the acoustic cavitation phenomenon, which involves the creation of bubbles and their subsequent rupture, which results in the release of bioactive substances. This rupture depends on the extraction circumstances.^86^ The ultrasound-assisted extraction methods use sound waves with a frequency range of more than 20 kHz and sound intensities of 5–1000 W/cm^2^. Some studies have reported using ultrasonic water baths, whereas others have employed ultrasonic generator probes. In the case of an ultrasonic probe, continuous ultrasonic waves are transmitted when the probe is directly inserted into the suspension.^87^ With 5 min or less, ultrasonic power is provided at least 100 times greater than the bath.^88^ On the other hand, in ultrasonic bath systems, the sample is exposed to ultrasonic radiation indirectly through the walls.^89^ In earlier research, methanol, acetone, ethanol, ethyl acetate, aqueous methanol, and water were used as solvents for phenolic and antioxidant component extraction from pomegranate peel.^90,88^

In accordance with the intended solvent/solid ratio, dried pomegranate peel powder (PPP) was combined with a solvent to create the sample mixture for the extraction procedure.^91^ According to the experimental plan, the samples were placed in an ultrasonic bath (Figure 3D) at a specific frequency, temperature, and duration.^92,93^ The ultrasonic probe (Figure 3D) includes using a probe sonicator to extract dried peel powder diluted with solvent at different amplitudes and duration levels, as required. The mixture’s temperature inside the extractor is measured using a thermocouple linked to the sonicator system.^94^

Variables such as pressure, temperature, intensity, wave frequency, surface tension, and solvent viscosity tend to impact the extraction process.^95^ This technique has several benefits over conventional extraction methods, including quick extraction time, a high rate of extraction, less solvent requirement, and ease of usage.^96^

#### Microwave-Assisted Extraction (MAE)

In the region of the electromagnetic spectrum between the radio frequency and far-infrared (IR), microwaves are high-frequency electromagnetic waves with wavelengths between 1 mm and 1 cm and frequencies between 0.3 and 300 GHz..^97^ The solvent was introduced after the dry samples were placed in extraction containers. The system was turned on after the container had been capped. The sensor precisely measured the temperature (Figure 3C). The heating appliance automatically turned off until the temperature fell after reaching the required temperature.^98^ Microwave-assisted extraction was effective because it can heat a matrix internally and externally without creating a thermal gradient. Microwave energy is strongly absorbed by molecules with permanent dipole moments, including phenolic compounds and ionic solutions. In addition, microwaves superheat the water molecules within the sample, which encourages breakdown and improves the retrieval of target elements from the matrix.^70^

A cavity magnetron produces electromagnetic waves to initiate microwave-assisted processes. Radiation waves interact with plant tissues, cell walls, and byproducts in the plant matrix. The plant matrix loses moisture due to electromagnetic energy, resulting in plant cell walls being subjected to pressure at the cellular and subcellular levels, which induces the swelling of plant cells. Eventually, the swelling causes structural modifications in the plant matrix, encouraging enhanced mass transfer of solutes due to cell breakdown. In turn, this promotes the leaching of phytochemicals from the plant’s cellular matrix into the extractant during the process.^99^ In one study, multiple solvent/peel ratios were created using a sample of powdered pomegranate peels, and the extracts were gathered for a specific period. Using different microwave powers and solvent/peel ratios, the difference in the extraction yield during the extraction process was examined. The following five solvents were used in each of the 13 experiments: water, 50 and 70% aqueous ethanol, and 50 and 70% aqueous methanol. Compared to ultrasound-assisted extraction, the microwave-assisted extraction method produced an around 1.7 times higher yield while taking less time to complete.^100^

#### Enzyme-Assisted Extraction (EAE)

The fundamental principle entails hydrolyzing food materials with an enzyme acting as a catalyst under ideal extraction conditions to liberate bioactive components. This approach aids in releasing bioactive substances like polyphenols, and other phytochemicals which are present within the cells but are complex to remove.^72^ To react at a specific pH, temperature, time, and enzyme dosage, dried samples were placed in a conical flask with a particular volume of distilled water (Figure 3B). Deactivation of the enzyme complex occurs, the mixture is filtered through four layers of gauze, and the procedures, as mentioned earlier, are repeated on the insoluble residue.^101−103^ Pectinase, protease, and cellulase are the enzymes employed in the literature, and the temperature ranged from room temperature to 45 °C. After enzymatic pretreatment, the hydrolyzed peels undergo extraction utilizing green technologies, including high pressure and ultrasound.^104^ Researchers investigated the synergistic use of two unconventional extraction techniques, microwave-assisted extraction (MAE) and enzyme-assisted extraction (EAE), using a cellulolytic enzyme preparation to effectively recover phenolics from pomegranate peels using 30% ethanol as the solvent. Using enzyme microwave-assisted extraction, more phenolic compounds with more significant antioxidant potential were successfully extracted with less solvent use in less time.^105^

#### Food Applications

Pomegranate peel has potential applications in the development of value-added products, owing to its high nutrient content and phytochemical characteristics. To replace the usage of synthetic antioxidants, pomegranate peel and extracts with antioxidant and anti-food-borne-pathogen bacterial properties may serve as an excellent choice, thereby preserving the quality and extending the shelf life of food products.^106^ Furthermore, adding pomegranate peel waste, in the form of either dried powder or its extract, acts as an enhancer of food products’ nutritional and organoleptic characteristics.^2^ The use of pomegranate peel in various formulations, including meatballs, beef burgers, muffins, cakes, cupcakes, biscuits, curd, fermented milk beverages, edible oils, and as a preservative, has been the subject of numerous research studies and experiments (Table 4).

**Table 4:** Utilization and Application of Pomegranate Peel in the Formulation of Value-Added Products

**Products**	**Formulations**	**Results and Findings**	**Ref**
**Meat Products**			
Goat meatballs	Lean goat meat, PPP, clove essential oil, oregano essential oil, refined vegetable oil, condiment paste, dry spice mix, and refined wheat flour.	Treated samples had considerably higher mean fat values and fiber percentages as compared to control samples.	(107)
Chicken meat patties	Chicken meat, sodium chloride, sodium tripolyphosphate, sodium nitrate, spice mix, condiments, breadcrumbs, water, egg liquid, fat, PPP, PPP aqueous extract, pomegranate aril baggage powder, pomegranate aril bagasse powder aqueous extract, and butylated hydroxytoluene.	Compared to pomegranate aril bagasse powder treated samples, PPP had a much higher phenolic content. During refrigerated storage, the TBA value of both control as well as treated patties rose dramatically. Nonetheless, during storage, the TBA values of PPP and aril bagasse powder were considerably lower than those of the control samples. The total plate count and psychrotrophic count of treated samples increase at a slower rate than that of control samples.	(108)
Refrigerated meatballs	Beef, breadcrumbs, onion powder, garlic powder, black pepper, cumin, coriander, salt and water, crude PPP, and nano-PPP.	The crude peel’s FRAP, total phenolic, flavonoid, scavenging activity, and reducing power increased after being ground. Crude and nano-PPP were added to the meatball, which prevented the development of volatile nitrogen, peroxide, and TBARS, thus preserving the sensory qualities for a cold storage period of 9 days.	(109)
Beef burger	Lean meat, fat tissues, sodium chloride, starch, garlic, onion, spice mixture, water, dried PPP.	Moisture content showed a downward trend with increased PPP concentration. Post refrigeration period of 12 days, the protein level of beef burger samples having pomegranate peel concentrations of 2 and 3% was relatively higher, at 14.33 and 14.77%. The considerable difference in TBARS values of samples with 1, 2, and 3% PPP and that of the control sample revealed the beneficial effect of pomegranate peel as a natural antioxidant source.	(110)
Refrigerated minced beef meat	Beef, pomegranate peel ethanolic extract, butylated hydroxytoluene, oil.	Samples treated with ethanolic extract of pomegranate peel experienced a considerable reduction in primary as well as secondary oxidation. Ethanolic extract of 1% concentration received the highest scores concerning organoleptic attributes (color, odor, and overall acceptability).	(111)
**BakeryProducts**			
Muffin cakes	Wheat flour, egg, sugar, corn oil, milk, PPP, and baking powder.	A significant increase in the total dietary fiber upon substitution with PPP. Control recorded 2.36%, and the PPP substituted sample ranged from 2.80% to 6.48%. Compared to the control muffins, all levels of PPP muffins exhibited significantly greater total phenolic content and antioxidant activity. The drop in the crumb and crust Hunter L and b values increases with the amount of PPP added.	(112)
Cakes	Wheat flour, soybean flour, PPP, baking powder, sugar, butter, eggs, and vanilla essence.	The crude fiber and ash content of all types of value-added cakes increased from 2.23 to 3.03% and 1.81 to 2.15%, respectively, as the proportion of PPP substituted increased. The control sample had an overall acceptability of 7.40, that is, liked moderately, whereas the cakes supplemented with wheat, soybean flour, and PPP at levels of 85:10:5, 82.5:10:7.5, and 80:10:10 received the score of 7.80, 7.74, and 7.96, respectively, falling into the “liked very much” classification.	(113)
Cupcakes	Wheat flour, PPP (5, 10, 15 and 20%), sugar, shortening, fresh egg, milk powder, baking powder.	Cupcakes with 20% supplementation recorded the highest value of ash (1.92%). The highest value for the taste was observed in cupcakes supplemented with 5% PPP. Overall acceptability exhibited a downward trend with regard to the subsequent increase in the concentration of PPP.	(114)
Biscuits	Wheat flour, margarine, sugar, salt, baking powder, and PPP.	The values of antioxidant activity, total phenolic content, soluble, insoluble, as well as total dietary fiber were observed as an increase in PPP. The flavor of biscuits made with 18% PPP was more acidic and bitter, which was thought to cause the fall in sensory scores.	(115)
**DairyProducts**			
Curd	Curd, PPE (dried powder), whey protein concentrate, skim milk powder.	With the successive increase in PPE concentration, the overall phenolic content and antioxidant activity of curd increased. However, sensory qualities deteriorated with a further rise in PPE concentration. PPE made curd resistant to microbial count development, pH fluctuations, and whey syneresis during storage period.	(116)
Fermented milk beverage	Milk, standard starter culture containing *Streptococcus thermophilus* and *Lactobacillus delbrueckii* subsp. *bulgaricus*, functional strains, *Lactobacillus plantarum*, and *Bifidobacterium longum* subsp. *longum*, PPE.	The antioxidant activity of the fermented milk beverage FMPO 300 (300 mg/mL) was higher than that of FMPO 150 (150 mg/mL). *In-vivo* research revealed that rats given a functional milk beverage for 30 days showed significantly lower levels of triacylglycerol, LDL cholesterol, and total cholesterol. Also, they had higher HDL cholesterol levels.	(117)
**Oil Products**			
Edible oil	Sunflower, soybean, and corn crude oils, PPE.	Compared to the negative controls (without antioxidant) and the synthetic antioxidant TBHQ-200, in all of the test oils, the pomegranate peel methanolic extract at varying concentration levels showed better antioxidant potential.	(118)
**Preservatives**			
Meat Products	Mutton ribs, spice mixture, condiment mixture, table salt, fat, and pomegranate rind extract.	Even though they were much lower across all storage intervals in the products treated with pomegranate rind extract, the free fatty acid (FFA) levels significantly rose from day 0 to day 21. Over the course of storage, the TPC of the products treated with pomegranate rind extract increased noticeably, and the values were continuously lower than the control. Pomegranate rind extract-treated products significantly outperformed controls regarding appearance and color between the 14th and 21st storage days.	(119)

#### Meat Products

Kumar et al.^107^ investigated how including PPP and essential oils affects the sensory aspect and the proximate composition of goat meatballs. Five distinct emulsions were prepared in total. PPP, as well as essential oils, were absent in the control; however, treatments were made incorporating PPP at a level of 3% along with the addition of clove essential oil, oregano essential oil, and their combination at levels of 0.25%, 1%, and 0.125% clove + 0.50% oregano oil in the respective treatment samples. With the advancement of time, a significant drop in the mean values of appearance was observed in the treatment group but not in the control group. This was attributed to oxidative rancidity, as suggested by an increase in the levels of thiobarbituric acid (TBA) and microbiological counts with prolonged storage. Although none of the treated samples drastically differed from one another, the average moisture percentage varied from that of the control group. No significant differences were observed between the control and samples in terms of fiber percentage and ash content.

Sharma and Yadav^108^ conducted a study to determine how the addition of bagasse powder, pomegranate peel, and their extracts affected the quality of chicken meat. Sodium tripolyphosphate, sodium chloride, spices, sodium nitrite, condiments, fat, water, egg liquid, and breadcrumbs were added in adequate proportions to the control samples of beef patties. In contrast, the treated samples were supplemented with pomegranate peel, bagasse, butylated hydroxytoluene (BHT), and aqueous extracts. Furthermore, the patties were kept in a refrigerator at a temperature of 4 to -2 °C, and their physical, chemical, and microbiological qualities were examined every 4–16 days of storage. The addition of BHT, pomegranate peel, bagasse, and their extracts significantly increased the phenolic content of chicken meat. The cooking yield and emulsion stability of patties treated with pomegranate aril bagasse powder were appreciably superior to those of the control. Compared to the control group, the treated samples had a noticeably higher ash content. This increase was due to the greater ash levels in the pomegranate bagasse powder and PPP. The thermophilic count, psychotropic count, and total plate count (TPC) rose considerably with longer storage times. Owing to the inhibitory effect of bioactive and phenolic substances, pomegranate byproducts and extract-treated patties proliferate relatively slowly.

ElBeltagy et al.^109^ studied the categorization of nano-pomegranate peel from physical, chemical, and antioxidant perspectives and its effect on refrigerated meatballs’ lipid oxidation. The dried pomegranate peels were pulverized and passed through a 50-mesh sieve. Pomegranate peel nanoparticles were created in a planetary ball mill and separated into two groups, NPP45 and NPP90, based on the amount of time they were ground. A homogeneous mixture of meat samples was prepared and divided into five proportions. The first portion was used as a control, and the subsequent three samples contained 0.5% CPP, 0.5% NPP45, and 0.5% NPP90. The fifth group was used as a reference, with the addition of 0.1% BHT. Analysis of the meatballs was performed at intervals of 3 days. In general, when the particle size of pomegranate peel was reduced by milling for 45 min (NPP45) and 90 min (NPP90), their DPPH-scavenging activities increased by 15.55% and 20.57%, respectively, and their FRAP increased by 20.37% and 35.18%, respectively. Incorporating crude/nanosized peels into the meatballs prevented the development of volatile nitrogen, peroxide, and thiobarbituric acid (TBARS). It reduced their levels while preserving the sensory qualities of the meatballs for up to 9 days of cold storage.

Abdel Fattah et al.^110^ experimented to determine how adding PPP at different ratios influenced the prepared beef burger’s ability to maintain quality and safety throughout a 12 day storage period at a temperature of 4 °C. Dried pomegranate peel was incorporated into the beef burger recipe at ratios of 1, 2, and 3%. The PPP-incorporated samples did not exhibit an appreciable pH variation. Significant improvements in the TBARS values of the samples containing 1, 2, and 3% PPP demonstrated the advantages of using PPP as a natural source of antioxidants. Results showed low TBARS readings at the start of the storage period, and during the post-storage period, the samples recorded values less than the critical limit, that is, 0.9 mg malonaldehyde/kg sample, and control samples recorded values higher than the critical limit, that is, 1.292 mg malonaldehyde/kg. After 3, 6, 9, and 12 days of refrigeration, the total bacterial count of the control sample without PPP increased considerably. It started at 3.32 log cfu/g at time zero and increased to 3.79, 4.23, 5.17, and 5.32 log cfu/g at 3, 6, 9, and 12 days, respectively.

Fourati et al.^111^ evaluated the relationship between *Punica granatum* peel extract and lipid/protein oxidation, as well as the sensory characteristics of refrigerated minced beef meat. The ethanol extract of the pomegranate peel was added to 200 g of ground beef at concentrations of 0.1, 0.5, and 1%. The fourth sample was prepared by using butylated hydroxytoluene (100 mg/kg). The fifth sample was used as a control devoid of any antioxidant source, and at 0, 3, 7, 14, and 21 days into the storage period, samples were collected. The aerobic plate count of the control sample showed a steep increase, reaching the minimum spoilage limit. In contrast, the samples containing 0.5% and 1% ethanolic extracts of pomegranate peel exhibited psychotropic counts as well as aerobic plate counts below the detection limits until 21 days of storage. The peroxide values of the extract samples were considerably lower than those of the control samples. Until the 14th day of the storage period, the samples containing the extract maintained the stability of their sensory attributes, which were later significantly reduced due to spoilage.

#### Bakery Products

The effects of adding pomegranate peel to muffin cakes on their chemical, physical, and nutritional attributes were studied by Topkaya and Isik.^112^ By replacing 5%, 10%, and 15% wheat flour with PPP, the effects of adding PPP to muffin cakes were examined. Compared to the control muffins, the muffins incorporated with 5%, 10%, and 15% PPP had total dietary fiber quantities that were 1.19, 2.10, and 2.75 times higher, respectively. A significant increase in the amount of PPP added to the muffin formulations was accompanied by noticeable increases in the magnesium, calcium, and potassium contents of the muffins. Compared to the controls, the antioxidant activity levels of the samples with 5%, 10%, and 15% PPP were 10.34, 22.23, and 28.47 times higher, respectively, while their total phenolic content was 3.08, 4.88, and 6.99 times higher.

Tharshini et al.^113^ evaluated cakes enriched with soybean and PPP in terms of organoleptic and chemical properties. Three samples were prepared by the addition of wheat, soybean flour, and PPP in the following ratios: 85:10:5 (I), 82.5:10.5:5 (II), and 80:10:10 (III), respectively, whereas the control sample was made entirely of wheat flour. The control sample received a color score of 7.50 and came under the “liked very much” category. On the other hand, Samples I and II received a score of 7.80, while Sample III received a score of 8.00, falling under the “liked very much” category. The score of 7.30, received by the control sample concerning taste, jumped considerably to scores of 7.90 (I) and 8.10 (III), thus falling under the “liked very much” category. Samples I, II, and III showed dietary fiber contents of 8.29%, 8.67%, and 9.07%, respectively, which were noticeably higher than that of the control sample with a dietary fiber content of 7.51%. A similarl outcome was observed for the mineral content.

Gdallah et al.^114^ employed PPP to prepare cupcakes supplemented with high dietary fiber. The effects of adding PPP to wheat flour substitutions at distinct ratios (5%, 10%, 15%, and 20%) were determined. When wheat flour, which had a dietary fiber content of 0.99%, was compared, PPP had a value of 12.12%. The moisture content of the cupcakes significantly increased after the addition of PPP. This increase was observed within the 13.25%–11.33% range for the cupcakes substituted with 5%–20% PPP, while the moisture content of the control sample was 9.84%. Regarding appearance values, there were no appreciable differences between the control sample and cupcakes substituted with 5% and 10% PPP.

Urganci and Isik^115^ studied the impact of different ratios of pomegranate peel on the chemical, physical, and sensory attributes of biscuits. To prepare the biscuits, wheat flour was substituted with PPP at 6%, 12%, and 18%. No considerable differences were observed between the control and PPP-substituted samples with respect to the crude ash, fat, and protein content. With an increase in PPP substitution, a significant increase in soluble and insoluble fibers was observed. Adding PPP reduces the hardness value, which is related to the force required to shatter the biscuits. The hardness value of the control sample was 9.62 ± 1.52 N; however, the results for the sample with 18% substitution were 4.12 ± 0.98. PPP substitution considerably darkened the color of the biscuits, with the brightest biscuit being the control sample.

#### Dairy Products

Sandhya et al.^116^ examined the effect of integrating PPE on the functional properties and shelf life of curds. After pretreatment of the pomegranate peel aqueous as well as ethanol extract, both were prepared in two concentrations, that is, 1:15 and 1:60. The antioxidant activities of both the extracts were evaluated. The ethanol extract exhibited higher levels of antioxidant activity and was thus selected for powder preparation. PPE was incorporated using six distinct levels of whey protein concentrate-70 (2, 3.5, 5, 10, 15, and 20%) and five distinct levels of skim milk powder (3.5, 5, 10, 15, and 20%). Both the antioxidant activity and total phenolic content of the curd showed an upward trend, with a corresponding increase in the level of PPE. Nevertheless, the sensory scores of all characteristics decreased with an increase in the extract level, possibly because of the astringent flavor of pomegranate peel. In the samples, the total microbial count decreased considerably compared to that in the control samples during storage. During the storage period, there was a marked decrease in the overall acceptability of the samples; however, the downward trend was more prominent and more rapid in the control.

PPE and lactic acid bacteria were used by Al-Hindi and Ghani^117^ to produce beneficial fermented milk beverages. The extract was added at concentrations of 150 and 300 mg/L. Preparation of the fermented milk beverage was performed using *Lactobacillus delbrueckii* and *Streptococcus thermophilus* as the starting cultures. In contrast, *Lactobacillus plantarum* and *Bifidobacterium longum* were used as potential probiotic strains. There was a significant decrease in the pH of the control and PPE samples with increasing storage times. DPPH and ABTS assays were used to determine the antioxidant activity during fermentation. After a storage period of 30 days, the 300 mg/L extract exhibited higher antioxidant activity; the second highest activity was observed at an extract concentration of 150 mg/L, and the control fermented milk showed the lowest activity compared to the samples incorporated with extracts. An *in vivo* study carried out on rats fed a functional milk beverage over 4 weeks showed the beneficial effects of the drink concerning a decrease in the level of total cholesterol triglycerides, thus exhibiting a positive impact on the lipid profile as compared to the control group, in which both PPE and probiotic bacteria were absent.

#### Oil Products

El-Hadary and Taha^118^ assessed the effect of methanolic PPE on the shelf life of edible oils under accelerated oxidation conditions. Vegetable oils were selected based on variations in polyunsaturated fatty acid composition. Six different treatments were applied to sunflower, soybean, and corn oil, and the mixture was kept at a temperature of 70 °C for 10 days. For the evaluation of oxidation defense activity against the negative control, devoid of any antioxidant, and the positive control, which used TBHQ at a concentration of 200 ppm as an antioxidant, methanolic extracts with concentrations ranging between 100, 200, 400, and 600 ppm were utilized. Different methanolic concentrations, along with the positive control, resulted in a marked decrease in peroxide values compared with the negative control. The peroxide values in sunflower, soybean, and corn oils were drastically reduced to 34, 20, and 6 mequiv/kg, respectively, when 100 ppm of pomegranate peel methanolic extract was added. These values were further lowered significantly when a 200 ppm concentration of pomegranate peel methanolic extract was employed; the corresponding amounts in sunflower, soybean, and corn oils were 24, 12, and 5 mequiv/kg, respectively. The peroxide values of the positive control were considerably similar to those obtained with the 200 ppm of methanolic extract. On the other hand, there was no significant difference between the 400 and 600 ppm pomegranate peel methanolic extracts. The conjugated triene concentrations in maize, soybean, and sunflower oils reached 10, 16, and 12 U, respectively, by the time the accelerated oxidation experiment was completed. The most striking result was the reduction of conjugated trienes in sunflower, soybean, and maize oils to 1.7, 5, and 2.5 U, respectively, using 600 ppm of pomegranate peel methanolic extract.

#### Preservative

A study by Dua et al.^119^ sought to ascertain whether pomegranate rind extract could replace the artificial antioxidants and preservatives present in meat products. Aqueous solutions of pomegranate rind extract at concentrations of 0.5, 1.0, and 1.5% were prepared. Varying concentrations of the pomegranate rind extract were applied after the products were developed, chilled, and treated before being aerobically packaged in LDPE pouches. They were tested for distinct quality criteria after 0, 7, and 21 days of refrigeration at a temperature of 4 ± 1 °C. From day 0 to day 21, the TBARS levels in all samples increased significantly; however, until the 14th day of storage, for both the control and the products combined with rind extract, these levels remained far below the allowable limit of 1 mg of malonaldehyde per kilogram. On the 21st day of storage, the products exceeded this limit. Free fatty acid levels significantly increased throughout storage; however, the values were much lower in the products containing 1% and 1.5% pomegranate rind extract. The total plate count in both the control and treated products showed a significant upward trend from day zero to the 21st day of storage. The microbial counts throughout the entire duration of the storage period for all items were below the permitted limits.

#### Toxicology, Safety, and Recommendations

Studies have found that PPP consumption may generate toxicity if it exceeds the threshold limits. It exhibited a favorable safety profile with an acute LD50 value of 731.1 mg/kg body weight. The potential toxicity of high doses of pomegranate peel and separate components at levels greater than 2000 mg/kg of body weight was examined in laboratory animals; however, when administered to BALB/c mice at doses up to 2000 mg/kg of body weight, PP galactomannan polysaccharides, which are known to possess cytotoxic properties against cancer cells, did not cause any observable toxic effects. A more recent study administered pomegranate ethanolic extracts orally to female rats at a concentration of 2000 g/kg of body weight. No toxicity was observed in the tested animals.^120^

Based on this study, oral administration of standardized pomegranate peel at a dose of up to 600 mg/kg of body weight per day did not cause adverse effects in rats and mice. However, higher concentrations were found to be increasingly toxic when brine shrimp were used as the test subjects. The study of the prevention of diarrhea observed an LD50 value of 1321 mg per kilogram of body weight in rats following intraperitoneal administration of pomegranate extract.^120^

In another study, diabetic rats were treated with PPE (100 mg/day). Rats with diabetes induced by streptozotocin (STZ) were treated with an extract derived from the pomegranate peel for 45 days. The results of the study showed a significant improvement (with a *P*-value of 0.05 or less) in the levels of serum glucose, cholesterol, and triglycerides, as well as in the levels of ALT and AST from the 15th day to the 45th day of the study when compared to the diabetic control group of animals.^121^

A previous study investigated the potential toxic effects of orally administered PPE on BALB/c mice. The results of this study indicated that repeated oral administration of the extract did not cause any toxic effects in terms of weight gain, food intake, behavioral or biochemical parameters, or irritation or inflammation of the oral cavity or epithelial cell layers of the tongue, larynx, and trachea. Furthermore, the administration of 0.5, 1.9, and 7.5 mg/kg of PPE did not cause death in any animal. None of the animals exhibited behavioral changes, and no signs or symptoms were observed. Biochemical studies revealed no glucose, cholesterol, ALT, or AST disturbances after PPE administration. The study also found that *Punica granatum* peel extract administered at a supratherapeutic dose of 7.5 mg/kg showed regular histopathological examinations with no inflammation. None of the animals developed any hematological or biochemical abnormalities, and all mice survived until the end of the study. Additionally, no allergic reaction was shown after 24, 48, and 72 h.^122^

The study found that pomegranate peel, a natural extract with excellent anti-inflammatory and antioxidant activities, was potent against autoimmune hepatitis (AIH) in a ConA-induced AIH mouse model. The results showed that PPE had substantial anti-inflammatory and protective effects on the occurrence and development of AIH. PPE reduced the mortality rate, transaminase levels, cytokine levels, and percentages of activated CD4+ and CD8+ T cells in the liver. The antioxidant activity of PPE may be associated with its ability to quench reactive oxygen species, remove oxygen free radicals, chelate metal ions, and inhibit oxidase. The potential mechanism of PPE in treating AIH may involve activating signal transduction pathways.^123^

Studies recommend that the consumption of pomegranate peel should be performed within safe limits to avoid toxicity. These values indicate that high doses of pomegranate peel may be toxic and that there is variability in the toxicologically relevant concentrations of pomegranate peel. Therefore, it is essential to carefully consider the dosage and composition of the extracts prior to consumption. Studies also suggest that PPP is a good source of total phenolics and possesses a high free radical scavenging activity, making it a highly effective antioxidant.

## Conclusion and Future Perspectives

Although pomegranate peel has historically been considered a waste material in the food industry, recent research has demonstrated its potential as a source of bioactive compounds that offer numerous health benefits. Studies have shown that pomegranate peel possesses various pharmacological properties, including antioxidant, anti-inflammatory, anticancer, and antimicrobial properties, making it a potentially valuable resource for the pharmaceutical and cosmetic industries. Extraction techniques play a crucial role in maximizing the potential benefits of pomegranate peels by efficiently obtaining specific components.

As consumer awareness of health benefits increases, there is a growing demand for natural products that have been shown to promote well-being. As a result, the trend toward natural products and health-conscious consumer behavior may lead to a surge in the demand for pomegranate peel as a source of bioactive compounds. Although pomegranate peel is considered a waste material, it has significant potential as a functional food ingredient because of its high nutritional composition and bioactive potential, which can be utilized by food manufacturers to create innovative products for health-conscious consumers. Additionally, exploring new extraction techniques and investigating the synergistic effects of combining pomegranate peel with other natural ingredients could further expand its application. This could lead to the creation of new ventures focused on the efficient use of pomegranate peel to produce functional foods and nutraceuticals, which could significantly impact the food industry in the future.
